# DNA copy number loss and allelic imbalance at 2p16 in lung cancer associated with asbestos exposure

**DOI:** 10.1038/sj.bjc.6605012

**Published:** 2009-03-31

**Authors:** E Kettunen, M Aavikko, P Nymark, S Ruosaari, H Wikman, E Vanhala, K Salmenkivi, R Pirinen, A Karjalainen, E Kuosma, S Anttila

**Affiliations:** 1 Health and Work Ability, Finnish Institute of Occupational Health, Topeliuksenkatu 41 aA, 00250 Helsinki, Finland; 2 Department of Pathology, Haartman Institute and HUSLAB, University of Helsinki and Helsinki University Central Hospital, Haartmaninkatu 3, 00140 Helsinki, Finland; 3 Laboratory of Computer and Information Science, Helsinki Institute for Information Technology, Helsinki University of Technology, Konemiehentie 2, 02015 Espoo, Finland; 4 Department of Tumor Biology, University Medical Center Hamburg-Eppendorf, Martinistr 52, 20246 Hamburg, Germany; 5 Work Environment Development, Finnish Institute of Occupational Health, Topeliuksenkatu 41 aA, 00250 Helsinki, Finland; 6 Department of Pathology and Forensic Medicine, Institute of Clinical Medicine, University of Kuopio and Kuopio University Hospital, P.O.BOX 1627, 70210 Kuopio, Finland; 7 Good Practices and Competence, Finnish Institute of Occupational Health, Topeliuksenkatu 41 aA, 00250 Helsinki, Finland

**Keywords:** lung cancer, asbestos exposure, genetic marker, allelic imbalance

## Abstract

Five to seven percent of lung tumours are estimated to occur because of occupational asbestos exposure. Using cDNA microarrays, we have earlier detected asbestos exposure-related genomic regions in lung cancer. The region at 2p was one of those that differed most between asbestos-exposed and non-exposed patients. Now, we evaluated genomic alterations at 2p22.1-p16.1 as a possible marker for asbestos exposure. Lung tumours from 205 patients with pulmonary asbestos fibre counts from 0 to 570 million fibres per gram of dry lung, were studied by fluorescence *in situ* hybridisation (FISH) for DNA copy number alterations (CNA). The prevalence of loss at 2p16, shown by three different FISH probes, was significantly increased in lung tumours of asbestos-exposed patients compared with non-exposed (*P*=0.05). In addition, a low copy number loss at 2p16 associated significantly with high-level asbestos exposure (*P*=0.02). Furthermore, 27 of the tumours were studied for allelic imbalances (AI) at 2p22.1–p16.1 using 14 microsatellite markers and also AI at 2p16 was related to asbestos exposure (*P*=0.003). Our results suggest that alterations at 2p16 combined with other markers could be useful in diagnosing asbestos-related lung cancer.

Lung cancer is the leading cause of cancer-related deaths, causing almost 1.2 million deaths per year worldwide (Parkin *et al*, 2005). Several risk factors are known to contribute to the outbreak of lung cancer, of which tobacco is by far the most predominant (Peto, 1994). However, exposures to occupational carcinogens, such as asbestos, are also well-established causal factors of lung cancer. Asbestos refers to a group of naturally occurring fibrous silicate minerals, which have been widely exploited in, for example, the building industry. Although banned in many countries, asbestos exposure continues to be a serious risk factor for lung cancer, because of the long latency period between initial exposure and outbreak of the disease (Consensus report, 1997; Henderson *et al*, 2004). Five to seven percent of all lung cancer cases are estimated to occur because of occupational asbestos exposure (LaDou, 2004). So far, probability based on epidemiological studies of lung cancer risk with a certain asbestos exposure level has been considered the best way to estimate whether, in individual cases, a lung tumour has been caused by the patient's earlier exposure to asbestos.

Several mechanisms have been proposed to be involved in asbestos-induced cytotoxicity and genotoxicity, the formation of reactive species of oxygen or nitrogen being particularly central (Xu *et al*, 1999; Unfried *et al*, 2002). Asbestos fibres have been shown to induce damage at both DNA and chromosomal levels, as well as abnormal chromosome segregation (Dopp and Schiffmann, 1998; Okayasu *et al*, 1999; Unfried *et al*, 2002). We were recently able to identify specific gene copy number and expression profiles in lung cancer of asbestos-exposed patients (Nymark *et al*, 2006; Wikman *et al*, 2007). In the earlier studies, six chromosomal regions were identified to be affected by both DNA copy number alterations (CNA) and gene expression changes, and differed between lung tumours of asbestos-exposed and non-exposed patients. One of the identified regions was 2p21–p16.3 (Nymark *et al*, 2006; Wikman *et al*, 2007). Furthermore, treatment with crocidolite asbestos fibres in the lung cell lines, A549 and Beas-2B, has been shown to result in altered gene expression at 2p22 (Nymark *et al*, 2007). Here we have further explored 2p22–2p16 by fragment analysis for detecting allelic imbalance (AI) and to localise the specific asbestos-targeted core region. Furthermore, CNA was studied in a large number of lung tumours by fluorescence *in situ* hybridisation (FISH). Particularly, we showed that low copy number loss and AI at 2p16 in lung cancer were significantly associated with patients' past occupational exposure to asbestos.

## Material and methods

### Lung tumours and normal lung tissue

Lung tumour samples from 205 patients were used in this study. The study population is presented in Table 1. Fresh-frozen lung tumour samples (*n*=81) have been obtained from patients operated during 1990–1996 at the Department of Thoracic and Cardiovascular Surgery of the Helsinki University Central Hospital. The informed consents of these patients were obtained and they have been personally interviewed for their work history. The Ethical Review Board for Research in Occupational Health and Safety, and the Coordinating Ethical Review Board, Helsinki and Uusimaa Hospital District (75/E2/2001) have approved the study protocol and the collection of fresh-frozen lung tumour samples and personally interviewed data.

Formalin-fixed paraffin-embedded (FFPE) samples of lung tumours (*n*=124) were received from different Hospitals in Finland. These patients' pulmonary asbestos fibre counts had been analysed at the Finnish Institute of Occupational Health as a part of earlier exposure assessment. The National Agency for Medicolegal Affairs (4476/33/300/05) permitted the use of tissue samples originally obtained for histopathological diagnosis, and the Ministry for Social Affairs and Health (STM/2474/2005) permitted the collection of patient information for this study.

The pulmonary asbestos fibre counts of all the patients were analysed by electron microscopy with energy dispersive spectrometry (Karjalainen *et al*, 1993). We considered a patient to be asbestos-exposed if the fibre count was ⩾1.0 million fibres per gram of dry weight, which is usually considered as a sign of occupational asbestos exposure and to be associated with increased risk for lung cancer (Karjalainen *et al*, 1993, 1994). The median fibre count among the exposed patients was 6.6 million g^−1^ (range 1.0–570 million g^−1^). Furthermore, asbestos-exposed patients with fibre counts of ⩾5 million fibres per gram were considered as highly exposed. It has been reported that the background level of asbestos in the general population may rise up to 1.0 million fibres per gram (Miserocchi *et al*, 2008). To highlight the contrast between the exposed and non-exposed, we considered patients with fibre counts of ⩽0.5 million fibres per gram to be non-exposed. Specimens obtained from patients with a fibre count between 0.5 and 1 million fibres per gram were not collected. In addition, morphologically normal lung from 27 of the lung tumour patients were studied for AI. The 27 patients included in the AI study have been studied earlier by cDNA microarrays, and among this group there were non-exposed patients and highly asbestos-exposed patients with pulmonary fibre count ⩾5.0 million fibres per gram (Nymark *et al*, 2006).

### Fluorescence *in situ* hybridisation

**Figure 1:**
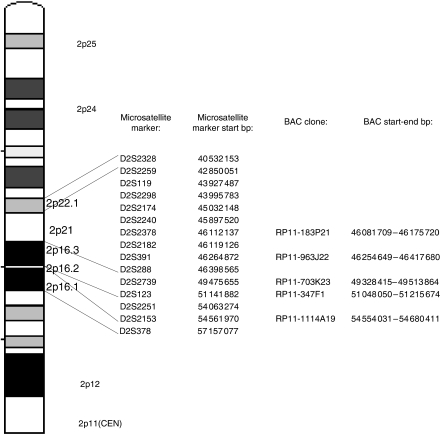
P arm of chromosome 2 showing microsatellite markers and bacterial artificial chromosome (BAC) clones used in fragment analyses and in fluorescent *in situ* hybridisation. Start and end base pairs are presented according to the University of California Santa Cruz (UCSC) genome browser.

Bacterial artificial chromosomes (BAC) from the clones RP11-1114A19, RP11-347F1, RP11-703K23, RP11-963J22, and RP11-183P21 were used as FISH probes. The probes are shown in Figure 1, together with the corresponding microsatellite markers. The BAC clones were obtained from BACPAC Resources (CHORI, Oakland, CA, USA). DNA was extracted using Qiagen plasmid midi kit (Qiagen, Valencia, CA, USA) and labelled by nick-translation with either biotin-14-dATP (Invitrogen Life Technologies, Carlsbad, CA, USA) (Kettunen *et al*, 2000) or Spectrum Green dUTP using a Nick Translation kit (Vysis Inc./Abbott Molecular Inc., Downers Grove, IL, USA). For the centromere of chromosome 2, either a Spectrum Orange-labelled CEP2 DNA probe (alpha satellite; Vysis, Inc./Abbott Molecular, Inc.) or a rhodamine-labelled chromosome 2 satellite probe (Qbiogene, KREATECH Biotechnology BV, Amsterdam, The Netherlands) was used. Both are referred to as CEP2 in the following.

A total of 87% of the FFPE tumours were sampled and put onto the tissue microarrays (TMA), and 13% of the FFPE tumours were studied as biopsy section slides. Before fluorescence microscopic analysis, serial sections of TMAs and biopsies were stained with haematoxylin and eosin for identification of tumour cells. After paraffin removal of TMA and biopsy section slides, and hydration of fresh-frozen tissue slides, the samples were treated in 0.01 M citrate, pH 6.0 at 80°C for 2 h (Chin *et al*, 2003). For digestion, we used DigestAll3 (Zymed Laboratories Invitrogen Immunodetection, San Francisco, CA, USA) at 37°C for 10 min. Denaturation, dehydrations, and hybridisation were carried out as described earlier (Kettunen *et al*, 2000; Andersen *et al*, 2001). Post-hybridisation washes for directly labelled BAC probes and for CEP2 were carried out according to manufacturer's instructions. Biotin-conjugated BAC probes were washed as described earlier (Kettunen *et al*, 2000), using fluorescein–avidin D and biotinylated anti-avidin D (Vector Laboratories Inc. Burlingame, CA, USA) for visualisation. The slides were counterstained with diamidino-2-phenylindole (DAPI) to identify tumour cells by morphology in fluorescence microcopy.

**Figure 2:**
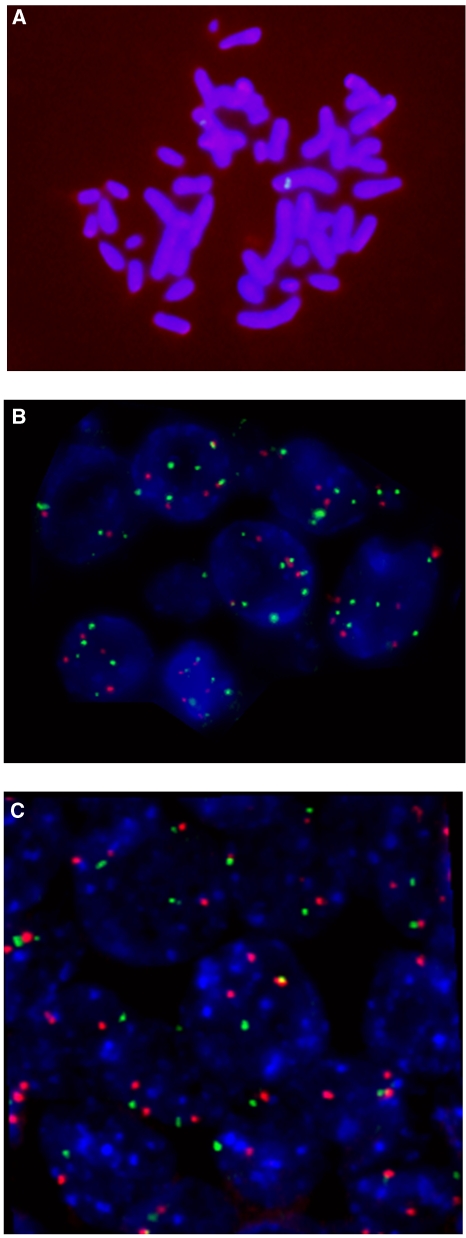
(**A**) An example of a metaphase showing hybridisation signals of the RP11-703K23 probe in chromosome 2. (**B**) Lung tumour cells hybridised with the CEN2 centromeric probe (red) showing diploid to tetraploid cells and with the RP11-963J22 probe (green) showing gain of DNA at 2p21. (**C**) Lung tumour cells hybridised with the CEN2 centromeric probe (red) showing diploid to tetraploid cells and with the RP11-703K23 probe (green) showing loss at 2p16.

All the BAC probes were hybridised on metaphase chromosomes to ensure correct localisation at 2p (Figure 2A). FISH hybridisations were analysed using a Zeiss Axioplan fluorescence microscope (Zeiss, Jena, Germany). To calculate the locus-specific copy number changes in each case, we divided the copy numbers of each probe by the mean count of centromere 2.

For each specimen, at least 100 cells were scored wherever possible. Owing to the large number of overlapping cells, we were able to count only 50–100 cells in 19% of the TMA specimens, and 30–50 cells in 12% of the TMA specimens and in 35% of the biopsy specimens. The mean signal count was calculated for each specimen. The FISH slides were divided into two sets containing specimens from both asbestos-exposed and non-exposed patients, and were analysed by two persons independently. Tissue cores with normal lung or lymph node material were used as negative controls in the TMA slides. In addition, lymphocytes or other non-cancerous cells showing two signals served as internal controls in each specimen.

To obtain locus-specific CNA at 2p in each case, we calculated the ratio between the mean signal count of each BAC probe and the mean signal count of CEP2. It has been reported that approximately half of all lung tumours are polyploid (Mitelman *et al*, 2009). In a case, in which the genome of the tumour cells is triploid, the fourth copy of locus DNA would show as a 33% increase in DNA copy number compared with the mean of centromeres. Thus, to be able to detect low CNA, we used the thresholds ⩾1.3 for gain and ⩽0.75 for loss of DNA.

### Fragment analyses for detection of allelic imbalances

DNA from snap-frozen normal lung tissue was extracted using QIAamp Tissue Kit (Qiagen). Sections (4 *μ*m) from snap-frozen lung tumour tissue specimens were stained with 1% toluidine blue–0.2% methylene blue solution for 20 s and dehydrated. The laser capturing procedure was carried out using Arcturus Veritas microdissecting device (Arcturus, Mountain View, CA, USA). Tumour cells were captured in CapSure Macro LCM caps (Arcturus, Mountain View, CA, USA) and DNA was extracted using Pico Pure DNA extraction kit (Arcturus).

Fragment analysis was carried out at 2p22–p16.1 (16.6 Mbp) using 14 microsatellite markers (Figure 1) to detect AI. Primers were labelled with FAM, HEX, or NED fluorochrome (TIB MOLBIOL Syntheselabor GmbH, Berlin, Germany or Applied Biosystems, Warrington, Cheshire, UK). The target sequences were amplified in tumour and corresponding normal lung tissue. PCR products of different sizes and fluorochromes were further combined and run with electrophoresis using 3100 Avant Genetic Analyzer (Applied Biosystems). The results were analysed with GeneMapper Analysis Software version 3.5 (Applied Biosystems). Allelic ratios were calculated between the two highest peaks with the expected fragment length for heterozygous markers in both tumour and normal DNA. Allelic imbalances were suggested when the ratio of the allelic ratios between the tumour and normal DNA was 2.0 or higher.

## Results

### Copy number alterations

Using FISH, we obtained the DNA copy numbers at 2p21–p16 in 55–156 lung tumours, depending on the probe and at centromere of chromosome 2 (alpha satellite) in 181 lung tumours. Locus-specific CNA was obtainable in 54–134 cases, depending on the BAC probe. The CEP2 signal count in lung tumour cells was on average 2.7 (range 1.6–5.7), varying between the lung tumour types as follows: adenocarcinoma of the lung (AC): 2.7; squamous cell carcinoma of the lung (SCC): 2.6; large cell lung cancer (LCLC): 3.0; and small cell lung cancer (SCLC): 2.6. Among the asbestos-exposed and non-exposed cases, the CEP2 mean signal counts were 2.7 (range 1.6–5.4) and 2.6 (range 1.6–5.7), respectively.

Locus-specific CNA results are shown in Table 2a. Both gains and losses of DNA were found (Figure 2B and C). In gains, the signals were evenly spread in the nuclei (Figure 2B).

The difference in DNA status between the exposed and non-exposed patients' lung tumours was tested at two stages. At first, we compared the frequencies of cases with losses, gains, and no CNA (Fisher's exact test or *χ*^2^-test). If *P* was <0.10, we continued by comparing the frequency of losses with the frequency of gains as well as the frequency of losses with the frequency of no CNA (Fisher's exact test or *χ*^2^-test). The RP11-703K23 probe at 2p16.3 showed association with asbestos exposure (*P*=0.09, Fisher's exact test) when comparing the difference between frequencies of losses, gains, and no CNA. Pair-wise comparisons, as explained above, of the number of cases with lost DNA and no CNA showed that losses at 2p16.3 were significantly associated with asbestos exposure (*P*=0.03, Fisher's exact test). Among the highly exposed patients (⩾5 million fibres per gram) the 2p16.3 losses were even more significantly associated with asbestos exposure (*P*=0.02, Fisher's exact test). An example of the lost RP11-703K23 signals is shown in Figure 2C. Gains and/or losses at 2p16 occurred in all histological types of lung cancer. When the frequencies of losses, gains, and no CNA were compared in different histological types, we found losses at 2p16 in the exposed patients with any histological tumour type, but the case numbers in each group were small and only SCC showed significant *P*-values. CNA in SCC were significantly associated with asbestos exposure at 2p16.2 (RP11-1114A19/CEN2, *P*=0.04, Fisher's exact test) and with high-level exposure at 2p16.3 (RP11-703K23/CEN2, *P*=0.02, Fisher's exact test).

In addition, the prevalence of probes that showed a CNA in each case at 2p16 (probes RP11-703K23, RP11-347F1, and RP11-1114A19) and at 2p21 (probes RP11-183P21 and RP11-963J22) was calculated. At 2p16, the prevalence of at least one probe loss was significantly increased in asbestos-exposed, particularly in highly exposed, in comparison with non-exposed cases (Table 2b). The difference was also significant between the exposed (100%, 2 out of 2 cases) and non-exposed (0%, 0 out of 7 cases) patients with SCLC (*P*=0.03, Fisher's exact test). No significant association was identified with asbestos when similar calculations were carried out for the gains (data not shown). It can be assumed that among the non-exposed of our study population no asbestos-induced cancers exist, whereas among the exposed patients the proportion of excess lung cancers because of asbestos exposure depends on the risk level in the group, and may be roughly 50% (Karjalainen *et al*, 1994). Thus, when combining the results of the three probes at 2p16, the specificity of copy number losses at 2p16 in detecting asbestos-related lung tumours would be 93%, but the sensitivity cannot be estimated because it is not known which of the lung cancers in the exposed group are asbestos-related.

### Allelic imbalance in 2p22.1–p16.1

**Figure 3:**
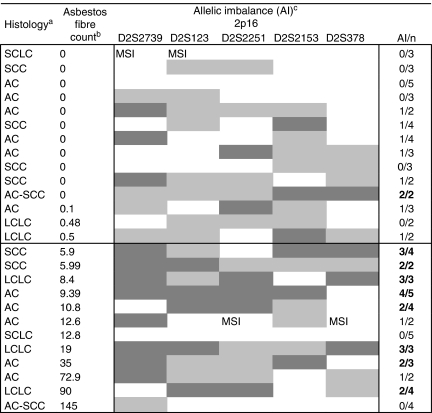
Significantly more allelic imbalances (AI) at 2p16 were found in asbestos-exposed patients' lung tumours compared with those of non-exposed (*P*=0.003, Fisher's exact test). ^a^AC, adenocarcinoma; SCC, squamous cell carcinoma; SCLC, small cell lung cancer; LCLC, large cell lung carcinoma; ^b^million g^−1^ dry lung; ^c^white, normal; light grey, no result; dark grey, allelic imbalance; MSI, microsatellite instability; bold when at least two markers showed AI.

A total of 27 lung tumours were microdissected for allele analyses and their microsatellite allelotyping profiles were compared with those in normal lung tissue of the same patients. Allelic imbalances at 2p22.1–p16.1 in the lung tumours of the asbestos-exposed and the non-exposed patients are shown in Table 3. The average proportion of informative microsatellite markers was 67% (range 41–83%). This agrees well with the reported heterozygosity rate of these markers. Microsatellite instability at 2p was observed in three cases: in two ACs, one from an asbestos-exposed and one from a non-exposed patient, and in one non-exposed SCLC. Although AI at 2p was observed to some extent in every lung tumour, we identified more AI in the lung tumours of asbestos-exposed (on average 52%) than in those of non-exposed patients (on average 32%). At 2p16.3 (D2S123), AI occurred in 63% of the asbestos-exposed and in 0% of the non-exposed patients' tumours (*P*=0.08, Fisher's exact test, Table 3). As the markers, D2S2739 and D2S2251, adjacent to D2S123 at 2p16 showed similar trends of asbestos association, we also looked at the five markers at 2p16 together (Table 3, Figure 3). Tumours having at least two informative markers out of five were included in the analysis. Allelic imbalances at 2p16 were scored for a tumour if at least two markers showed AI. We found significantly more AI in asbestos-exposed patients compared with non-exposed (*P*=0.003, Fisher's exact test; Figure 3).

## Discussion

We analysed 205 lung tumours for CNA at 2p and 27 lung tumours for AI at the same region. We showed that copy number loss at 2p16 is significantly associated with a patients' past occupational asbestos exposure. Particularly, we found that the prevalence of low copy number loss at 2p16 shown with three different FISH probes was significantly associated with asbestos exposure (*P*=0.04, *χ*^2^-test). In addition, AI at 2p16 was detected more frequently in the tumours of asbestos-exposed patients (*P*=0.003, Figure 3).

The chromosome count and structure in lung cancer cells often show great variation. Some aberrations in chromosome 2 have been detected in lung cancer, for example the amplification of the *MYCN* locus (2p24) in SCLC (Knuutila, 2004; Kim *et al*, 2006). Recently the fusion gene, *EML4*-*ALK*, resulting from an inversion at 2p21/2p23, was found in 3–7% of non-small cell lung cancer (NSCLC) (Soda *et al*, 2007; Koivunen *et al*, 2008). Furthermore, by array comparative genomic hybridisation it has been shown that 52% of NSCLC carried gained sequences at 2p21.1–p14 independent of histological type (Dehan *et al*, 2007). However, because the asbestos exposure burden of a given lung cancer patient has not usually been determined, the majority of earlier studies understandably lack association analyses between genomic findings and asbestos exposure status. Our earlier array studies on lung cancer showed that genomic alterations at 2p21–p16.3 may be related to asbestos exposure in lung cancer patients (Nymark *et al*, 2006; Wikman *et al*, 2007).

In this study, we have further characterised the asbestos-related genetic aberrations at 2p in a large group of lung cancer patients. We detected asbestos-associated loss and AI at 2p16 and localised the core region at 2p16.3–p16.2 (Table 2a).

Our results indicate that asbestos specifically targets regions such as on chromosome 2. Asbestos has been shown to cause hyperdiploidy, loss of chromosomes, and chromosomal breakage (Dopp and Schiffmann, 1998). Although the precise mechanisms and outcomes of asbestos-induced DNA damage have not yet been thoroughly established, the finding of DNA loss and AI at 2p16 in the lung tumours of asbestos-exposed patients is in accordance with the current knowledge on the behaviour of asbestos fibres. Furthermore, common fragile sites may be prone to replicative stress and increased frequency of AI. We have earlier detected an association between fragile sites and some of the asbestos-associated CNA (Nymark *et al*, 2006). The aphidicolin-sensitive common fragile site, FRA2D, is located at 2p16.2 between the markers, D2S123 and D2S2153, and seems to overlap with the region showing asbestos-associated loss (Schwartz *et al*, 2006). Some tumours, such as a cancer syndrome called the Carney complex (CNC), have shown both amplifications and deletions at 2p21-p16 (Matyakhina *et al*, 2003). The CNC syndrome is a genetically heterogeneous disease associated with multiple neoplasms, such as skin, cardiac, and breast myxomas, as well as endocrine tumours. The size of the CNA at 2p has been reported to vary from case to case and from cell to cell, indicating that 2p is indeed prone to variable alterations.

In our earlier work, the affected sequences in which the DNA copy number differed significantly between lung tumours of asbestos-exposed and non-exposed patients, was estimated to situate at 2p21–p16.3 (Nymark *et al*, 2006). Here we were able to more specifically localise the affected region to 2p16 (RP11-703K23). Alternation of normal and abnormal loci makes it challenging to identify typical asbestos-related alterations, particularly in a complex genome, such as in lung cancer, which often shows polyploid genome (Mitelman *et al*, 2009). Nevertheless, by comparing locus-specific probes with a centromere probe to obtain locus-specific CNA at 2p, we were able to identify asbestos-associated CNA, although the alterations occurred at low level. We anticipate that genomic alterations occurring at 2p may reflect distinct events in lung tumours, some related to asbestos exposure and some not. Independently of patients' asbestos exposure status, we detected frequent low copy number gains at 2p21, which suggests that 2p21 may harbour genes important to the development of lung cancer, such as the earlier mentioned oncogenic *EML4*-*ALK* fusion gene. It seems that, although chromosome 2 may generally be affected by gains in lung tumours, asbestos may cause breaks in the chromosome and thus result in additional alterations, such as losses.

In conclusion, we show that locus-specific copy number loss and AI at 2p16.3-p16.2 are associated with asbestos exposure in lung tumours, whereas low-level copy number gains at 2p21 are common in lung cancer in general. Probability based on epidemiological studies has for a long time been used as a method of indicating that asbestos exposure burden may be associated with an individual's lung cancer. We believe that the findings we describe here may benefit the diagnosis of asbestos-associated lung tumours, when combined with earlier identified asbestos-associated alterations in other chromosomal regions, such as 19p13 and 9q33.1 (Ruosaari *et al*, 2008; Nymark *et al*, 2009).

## Figures and Tables

**Table 1:** Summary of study population

	**Fluorescence *in situ* hybridization (FISH)**		**Allelic imbalance (AI)**
	**Formalin-fixed paraffin-embedded material**	**Surgical fresh-frozen material**	**Surgical fresh-frozen material**
	***n*=124**	***n*=81**	***n*=27^a^**
*Sex*			
Male/female	119/5	73/8	27/0
			
*Age*			
Mean (range)	65 (42–87)	62 (36–81)	62 (41–72)
			
*Histology of tumours and asbestos exposure status*			
AC exposed	23	16	5
AC non-exposed	14	18	6
SCC exposed	28	11	3
SCC non-exposed	19	17	4
SCLC exposed	11	1	1
SCLC non-exposed	9	4	1
LCLC exposed	7	4	3
LCLC non-exposed	3	3	2
Other LC exposed^b^	7	1	1
Other LC non-exposed^c^	3	6	1

Abbreviations: AC=adenocarcinoma; LC=lung cancer; LCLC=large cell lung carcinoma; SCC=squamous cell carcinoma; SCLC=small cell lung cancer.

a Same tumours as fresh-frozen material are included in the FISH study.

b Tumours consisted of three pleomorphic, three large cell neuroendocrine carcinomas, one adenosquamous carcinoma, and one undefined non-small cell lung cancer in FISH study, and one adenosquamous carcinoma in AI study.

c Tumours consisted of one pleomorphic and four adenosquamous carcinomas, one undefined non-small cell lung cancer, and three large cell neuroendocrine carcinomas, one combined with squamous cell cancer in FISH study, and one adenosquamous carcinoma in AI study.

**Table 2a:** The numbers (%) of highly asbestos-exposed (⩾5 million fibres), asbestos-exposed (1–<5 million) and non-exposed patients' lung tumours with lost (ratio <0.75), gained (ratio >1.3), or normal 2p DNA sequences

**Probe location**	**RP11-183P21**			**RP11-963J22**			**RP11-703K23^a^**			**RP11-347F1**			**RP11-1114A19**		
	**2p21**			**2p21**			**2p16.3**			**2p16.3**			**2p16.2**		
	***n*=82**			***n*=75**			***n*=134**			***n*=95**			***n*=54**		
	**Highly exp (*n*=31)**	**Exp (*n*=13)**	**Non-exp (*n*=38)**	**Highly exp (*n*=22)**	**Exp (*n*=15)**	**Non-exp (*n*=38)**	**Highly exp (*n*=41)**	**Exp (*n*=29)**	**Non-exp (*n*=64)**	**Highly exp (*n*=27)**	**Exp (*n*=20)**	**Non-exp (*n*=48)**	**Highly exp (*n*=18)**	**Exp (*n*=8)**	**Non-exp (*n*=28)**
*DNA status*															
Loss^b^	2 (6)	0 (0)	1 (3)	0 (0)	0 (0)	1 (3)	**6** (**15)**	**2** (**7)**	**1** (**2)**	**5** (**19)**	**3** (**15)**	**4** (**8)**	**2** (**11)**	**3** (**38)**	**3** (**11)**
Gain	9 (29)	4 (31)	14 (37)	14 (64)	5 (33)	17 (45)	2 (5)	4 (14)	6 (9)	2 (7)	3 (15)	8 (17)	2 (11)	1 (13)	1 (3)
Normal	20 (65)	9 (69)	23 (60)	8 (36)	10 (67)	20 (52)	33 (80)	23 (79)	57 (89)	20 (74)	14 (70)	36 (75)	14 (78)	4 (50)	24 (86)

Abbreviation: Exp=exposed.

a The probe providing best differentiation between the asbestos-exposed and non-exposed patients' tumours. Fisher's exact test between non-exposed and exposed (⩾1 million, *P*=0.09), and between non-exposed and highly asbestos-exposed (⩾5 million, *P*=0.02).

b The groups with the most significant difference are given in bold.

**Table 2b:** Numbers (%) of lung tumours showing loss at 2p16 according to asbestos-exposure level and extent of the lost DNA

**Number of probes^a^ showing loss at 2p16**	**Highly exp^b^ (*n*=41)**	**Exp^c^ (*n*=29)**	**Non-exp^d^ (*n*=71)**
0	32 (78)	24 (83)	65 (91)
1	5 (12)	3 (10)	4 (6)
2	4 (10)	1 (3.5)	2 (3)
3	0 (0)	1 (3.5)	0 (0)

Abbreviation: Exp=exposed.

a Including the probes RP11-703K23, RP11-347F1, and RP11-1114A19.

b Pulmonary fibre count ⩾5 million fibres per gram.

c Pulmonary fibre count 1–<5 million fibres per gram.

d Pulmonary fibre count ⩽0.5 million fibres per gram.

*P*=0.05, highly asbestos-exposed and asbestos-exposed *vs* non-exposed; *P*=0.04, highly asbestos-exposed *vs* non-exposed (*P*-values were calculated using *χ*^2^-test for the difference between at least one loss and no losses).

**Table 3:** Allelic imbalance (AI) in 14 microsatellite markers at 2p22.1–p16.1 in lung tumours of asbestos-exposed and non-exposed patients

		**AI/n (%)^a^**													
		**Microsatellite markers within the chromosomal region**													
		**2p22.1**	**2p21**								**2p16.3**		**2p16.2**		**2p16.1**
**Exposure**	**Asbestos fibre count^b^**	**D2S2328**	**D2S2259**	**D2S119**	**D2S2298**	**D2S2174**	**D2S2240**	**D2S2378**	**D2S2182**	**D2S391**	**D2S2739**	**D2S123**	**D2S2251**	**D2S2153**	**D2S378**
Asbestos-exposed *n*=13	33.4 (5.9–145.0)	2/11 (18)	5/9 (56)	5/10 (50)	4/10 (40)	3/5 (60)	5/10 (50)	7/8 (88)	1/3 (33)	4/7 (57)	8/11 (73)	5/8 (63)	4/7 (57)	3/8 (38)	4/9 (44)
Non- exposed *n*=14	0.06 (0.0–0.5)	1/8 (13)	3/7 (43)	1/8 (13)	3/9 (33)	3/7 (43)	4/10 (40)	4/10 (40)	3/6 (50)	3/7 (43)	3/9 (33)	0/5 (0)	2/9 (22)	3/8 (38)	1/10 (10)
*P* c		1.00	1.00	0.15	1.00	1.00	1.00	0.07	1.00	1.00	**0.17**	**0.08**	**0.30**	**1.00**	**0.14**

a Number of cases with AI/informative cases.

b Million g^−1^ dry lung; mean (range).

c *P* (Fisher's exact test); bold if the corresponding marker is presented in Figure 3.
